# Internationally Adopted Children from Non-European Countries: General Development during the First Two Years in the Adoptive Family

**DOI:** 10.1100/2012/375436

**Published:** 2012-07-31

**Authors:** Monica Dalen, Steinar Theie

**Affiliations:** Faculty of Educational Sciences, University of Oslo, P.O. Box 1161, 0318 Oslo, Norway

## Abstract

Internationally adopted children are often delayed in their development and demonstrate more behaviour problems than nonadopted children due to adverse preadoption circumstances. This is especially true for children adopted from Eastern European countries. Few studies have focused on children adopted from non-European countries. This paper presents results from an ongoing longitudinal study of 119 internationally adopted children from non-European countries during their first two years in Norway. Several scales measuring different aspects of the children's development are included in the study: communication and gross motor development, temperamental characteristics, and behaviour problems. The results show that internationally adopted children are delayed in their general development when they first arrive in their adoptive families. After two years the children have made significant progress in development. However, they still lag behind in communication and motor skills compared to non-adopted children. The temperamental characteristics seem very stable from time of adoption until two years after adoption. The children demonstrate a low frequency of behaviour problems. However, the behaviour problems have changed during the two years. At time of adoption they show more nonphysically challenging behaviour while after two years their physically challenging behaviour has increased.

## 1. Introduction

International adoptions started in Norway at the end of the 1960s, and today there are around 19 000 international adoptees in this country. Most of the children come from China, South Korea, Colombia, and Africa [1].

A high percentage of internationally adopted children were placed in institutions and orphanages during the first months of life. It is well documented that children placed in institutions at a very young age are exposed to a variety of negative preadoption factors. These factors may include neglect and malnutrition due to a lack of sufficient personnel to meet the needs of these children [2]. Institutionalization is associated with developmental delays and behaviour problems because institutional conditions are characterized by decreased opportunities for sensory exploration and social-emotional exchanges. As a result, the children may not be able to process and utilize sensory information to guide and regulate their behaviours effectively [3]. Children who spend extended time in institutions usually experience less environmental complexity and thus may be at greater risk for neurobehavioral dysfunction [4, 5]. This is especially true for children adopted from Romania and other Eastern European countries who have been placed in institutions with very low quality of care [6]. Children adopted from other countries like South Korea, Colombia, and China may have been less exposed to many of these risk factors, which might make them less vulnerable to delays in their development. There is a need for more research on children who have grown up under less adverse pre-adoption conditions than children adopted from Eastern Europe. 

In the present study we examine communication and motor development as well as temperamental characteristics and behaviour problems among internationally adopted children from non-European countries during the first years in their adoptive families.

When children are adopted internationally one would expect them to be more or less delayed in their overall motor development due to their previous restricted possibilities for practicing motor skills, social interaction, and general personal comfort. Many of these children might be at risk for developmental disabilities and delays and it is therefore important to monitor them from an early age [7]. Today there is a need for more research to completely understand the link between delays in the adopted children's functioning in different areas on arrival and in their later development. 

Findings have been quite consistent in indicating that individual temperamental characteristics such as high levels of emotionality from early age on have a clear prospective relationship to behaviour problems [8, 9]. However, temperament has less frequently been studied as a predictor of change in problem behaviour [10]. High temperamental shyness and emotionality consistently predict internalizing problems [11]. Externalizing problem behaviour, on the other hand, is related to high scores on activity level [12]. 

Temperament is often defined as inherited personality traits presented in early childhood and might be found among genetically similar population and species [13]. Internationally adopted children coming from the same continent might expose similar personality traits.

It is hard to describe children's temperamental characteristics before adoption since we have very little valid information on internationally adopted children's genetics. When they arrive in their new families, the adoptive parents are able to evaluate these characteristics as they appear in the child's behaviour. In this study we will examine these temperamental characteristics at time of adoption and again two years later. 

It is well documented that internationally adopted children exhibit more behaviour problems than non-adopted children [14, 15]. Different kinds of behaviour problems have been discovered in postinstitutionalized children [2, 6]. Some studies have shown that children with adverse pre-adoption experiences show increased rates of inattention, hyperactivity, attachment problems, and autism-like traits. Gunnar and Van Dulmen [2] documented that institutional privation per se is not associated with an increase in all types of behaviour and emotional problems. They indicate that the problems most often are limited to attention, thinking, and social problems. In this study we will examine both physically challenging behaviour and nonphysically challenging behaviour which could be classified as more externalized behaviour. 

All Internationally adopted children will experience a dramatic change in their lives during the adoption process. Their reactions to this change will naturally vary with their age at adoption and maybe even more with their earlier experiences in life. A child who has been in a foster home or with the biological mother for some time will have different reactions than a child who has spent the first year in orphanages without much personal contact with adults. Quite a few studies have shown that adopted children have a variety of emotional reactions on arrival due to the process they have gone through [16–18]. However, many of these reactions seem to disappear after three to six months after adoption. Very few studies have focused on what kind of impact such transition reactions may have on the children's later development. For some children a strong emotional reaction when being moved from a safe place to more unpredictable conditions can be very healthy and normal. Actually it can be an adaptive reaction which will help the child in the later attachment process with its new adoptive parents. It is therefore important to obtain information on children showing different intensity in transition reactions during the adoption process.

**Aims and Research Questions**

The present study is part of a longitudinal research project following 119 internationally adopted children from time of adoption through preschool and school years. In this article the focus is on the internationally adopted children's communication and motor development, temperamental characteristics, and behaviour problems from time of adoption until two years after adoption. One would expect the adopted children to have some delays in their motor and language development due to the adverse pre-adoption conditions many of them have been exposed to. For the same reason adopted children will also be vulnerable to developing some kind of behaviour problems. At the same time it has been well documented that adoption has a positive effect on children's development due to new and more stimulating environments. The specific research questions we address are as follows.

1. Do internationally adopted children catch up in communication and motor development from time of adoption until two years after adoption?
2. How do adopted children's temperamental characteristics and behaviour problems change from time of adoption until two years after adoption?
3. Which of the included variables explain most of the variance in the children's general development at two years after adoption?

## 2. Methods

The longitudinal study started in 2007 and followed internationally adopted children from time of adoption with interviews at different age stages, play observations, and tests. This article is based on interviews with parents focusing on internationally adopted children's development in different areas from age of adoption until two years after adoption. The study is part of a larger study following 1.159 Norwegian born children at the corresponding ages [19]. This gives unique possibilities to compare developmental trajectories in adopted and non-adopted children.

### 2.1. Participants

Selection criteria for participating in the adoption study were children adopted to Norway during 2007–2009. The child's age at adoption should be under two years, and the families were selected from central parts of east Norway. The adoptive families were first contacted with information from the Norwegian Directorate for Children, Youth and Family Affairs. A total of 178 families met the selection criteria and received information about the possibility to participate. A total of 119 families wanted to participate, giving a response rate of 67%. The 119 adopted children were compared to the total of 178 adopted children on relevant demographic variables. There were no significant differences between the children in the originally selected group and children in the participating group regarding gender, age of adoption, and country of origin. 

### 2.2. Procedures

Both the interviews at time of adoption and two years later were carried out in the adoptive family's private homes. The interview guides were computer based and consisted of two parts. The first part of the interview was guided by the interviewer. The second part was self-instructive and included more sensitive questions related to the child's development and the parent's personality. 

### 2.3. Measures 

#### 2.3.1. General Development

Parents completed the communication and gross motor scales of the 12-month and 24-month ages and stages questionnaire, 2nd edition (Norwegian version) [20, 21]. The ASQ is a screening test, and most children are expected to have high scores representing normative development. One item from each scale had been omitted; the omitted items were more liable to require trying out with the child, which might not have been possible for all participants at the time of completion. Items are scored 0 (not yet), 5 (sometimes), or 10 (yes) and scale scores are computed as mean item scores (after adjusting for one missing item according to the procedure in Janson [21] with higher scores representing more advanced development.

#### 2.3.2. Child Temperamental Characteristics

Child temperamental characteristics were measured by using the Buss and Plomin [13] EAS questionnaire on three dimensions: *emotionality* (the tendency to become aroused easily and intensely), *activity* (preferred levels of activity and speed of action), and *shyness* (the tendency to be inhibited and awkward in new social situations). Items are scored 1 (very typical) to 5 (very untypical); scale scores are computed as mean item scores after the reversal of some items. The Norwegian translation of the EAS questionnaire has previously been explored by Mathiesen and Tambs [22] in a sample of Norwegian children and shown to have sound psychometric properties. 

#### 2.3.3. Behaviour Problems

*Physically and Nonphysically Challenging Behaviour. *Parents completed a comprehensive item pool of child behaviours developed for the current project. We used the mean of frequency ratings for physically and non-physically challenging behaviours rated from 1 (never) to 7 (three times daily or more). The physically challenging behaviours included were: hits you, hits siblings (if applicable), hits other adults, pushes someone to get what he/she wants, pulls someone's hair, pinches someone, throws things at others, bites someone, and kicks someone. The non-physically challenging behaviours were being noisy, fussy, crying and having temper tantrums.

#### 2.3.4. Transition Reactions

Parents completed a scale measuring different transitional reactions they observed during the first month after adoption. This scale was composed in 1992 by Dalen and Sætersdal and has later been used in several adoption studies [16, 23–25]. The scale includes reactions related to sleep, anxiety, contact with parents, confidence, and attention seeking. Items are scored 1 (no reactions), 2 (some reactions), and 3 (strong reactions).

### 2.4. Statistical Analysis

To compare differences between the children's scores at time of adoption and two years later a paired sample *t*-test was used for scores on all subscales included in the study. Effect sizes were measured with Cohen's *D*. Cohen characterizes effects under  .2 as no effects, between  .2 and  .5 as small effects, between  .5 and  .8 as medium effects and over  .8 as large effects [26].

Within the adopted group, a series of repeated measures analysis of variance was applied to all subscales included with a 2 (Child's gender: girl, boy) × 2 (Child's adoption age: below and above 12 months) mixed design. 

Reliability of each scale was measured for internal consistency by using Cronbach's alpha coefficient (*α*). The mean interitem correlation was *α* = .78 both at time of adoption and after two years.

Partial correlation analysis (Pearson's *r*) was carried out for all variables, measuring scores at adoption and after two years with age at adoption as the confounding variable. 

Communication competence was used as the dependent variable in linear multiple regression analysis to study variables explaining the variance in children's functioning both at time of adoption and after two years. Age of adoption, gender, country of origin, transition reactions, motor development, temperamental traits, and behaviour problems were used as independent variables. Finally hierarchical multiple regression analysis was conducted for each of the measured variables (communication, gross motor development, temperamental traits, and behaviour problems) using scores after two years as dependent variables. Step  1 in the analysis included competence at age of adoption (autoregressors) on all scales as independent variables. Gender, age of adoption, and transition reactions were entered at Step  2. 

Preliminary analyses were conducted to ensure that the residuals were normally distributed and to avoid homoscedasticity and multicollinearity. 

## 3. Results

### 3.1. Sample Demographics

A total of 112 internationally adopted children participated in the study both at time of adoption and two years later. The children were adopted through three different adoption agencies in Norway and consisted of 57 girls and 55 boys. The age at adoption ranged from 4 to 22 months (*M* = 11.2, SD = 5.0) and the children were adopted from the following countries: China, South Korea, South Africa, Colombia, Ethiopia, the Philippines, Thailand, and India.  Table 1 gives an overview of these children. Strong transition reactions were found in 20 (18%) of the children while almost the same number showed no such reactions (19%). The rest of the group (62%) showed moderate reactions.

### 3.2. Comparisons between Time of Adoption and Two Years after Adoption

The adopted children's scores on communication, gross motor development, temperamental traits, and behaviour problems at time of adoption and two years later are shown in Table 2.

The adopted children have increased their skills in communication after two years compared to the time of adoption. This also applies to the children's gross motor development. The children's temperamental trait pattern has changed less during the two years. The children are showing more emotionality after two years than when they first arrived. Furthermore, the adopted children are showing more physically challenging behaviour two years after adoption. Many are hitting and biting parents. At time of adoption they demonstrated more non-physically challenging behaviour like having temper tantrums and anger without being physically abusive. Following the guidelines proposed by Cohen [26], two of the presented effect sizes would be interpreted as being median (communication and non-physically challenging behaviour). The effect sizes of the statistical significant development of the other characteristics are small. However, gross motor development and emotionality reach the levels of  .360 and  .310, which is noteworthy. T values for activity level and shyness are not significant and will not be discussed further in the text.

### 3.3. Comparisons of Groups within the Sample

The ANOVA analyses documented no significant interaction effect between gender and age of adoption in the children's scores on communication, gross motor development, temperamental traits, and behaviour problems either at time of adoption or two years on.

A significant effect of age at adoption was found for communication, *F* (1,105) = 4.396, *P* = .000; gross motor development, *F* (1,105) = 2.778, *P* = .001; physically challenging behaviour, *F* (1,111) = 2.318, *P* = .005; and non-physically challenging behaviour, *F* (1,105) = 2.172, *P* = .012. Children aged above 12 months at adoption score higher on communication and motor development. This group also scores higher on physically and non-physically challenging behaviour than children aged below 12 months at adoption.

Two years after adoption a significant gender effect was found for communication, F (1,107) = 5.396, *P* = .023 and physically challenging behaviour, F (1,108) = 7.180, *P* = .009. Girls score higher on communication while boys show more physically challenging behaviour. 

Because of the wide age range at adoption, age was controlled for in an analysis of correlation between scores on the different variables measured at adoption and two years later.  Table 3 presents partial correlations coefficients showing that age at adoption had a stronger impact on communication and gross motor development than on temperamental characteristics and behaviour problems.

A linear multiple regression analysis was performed to examine the variance in the adopted children's communication competence at time of adoption and two years later. The independent variables of gender and country of origin were recoded into dummy variables. The results are presented in Table 4.

The independent variables explain more of the variance in the children's communication at age of adoption (65%) than two years later (38%). Gender, age of adoption, and gross motor development explained most of the variance at time of adoption. Transition reactions, gross motor development, and physically challenging behaviour had the strongest impact on communication competence two years later. 

Results from hierarchical multiple regression analysis for all the variables measured in the study is presented in Table 5.

Step  1 in all of the analyses included competence at age of adoption (autoregressor). In Step  2 age of adoption, gender, and transition reaction were added as independent variables. Country of origin did not contribute significantly to the variance in communication either at time of adoption or two years later (see Table 4) and was therefore excluded from the hierarchical regression analysis.

The results show that the children's communication competence and gross motor development at time of adoption predicted to a fairly low degree of their performance in these areas two years after adoption. The children's temperamental traits and their behaviour problems at time of adoption seem to be better predictors of later development. 

## 4. Discussion

The aim of the present study was to explore development in internationally adopted children during their first two years in their new families. The results indicate that the children do develop significantly from age of adoption until two years after adoption both in communication and gross motor competence. However, compared to non-adopted children, they still lag behind in their development [27]. This was expected considering that many of the children have spent their first months of life in institutions with low quality of care and lack of possibilities for physical exploration [2, 6]. The significant increase in development can be explained both by the natural growth in age but also to new environmental conditions in the adoptive families. Many studies have documented the ability of adoptive parents to provide stimulating environments for their children in different developmental areas [28–30].

The children's temperamental characteristics did change in one of the measured areas; emotionality. The children showed more behaviour such as being aroused easily and intensely after two years. This can be explained by the dramatic change in life they have all been exposed to. Around 18 % of the children had a strong transition reaction at time of adoption often related to problems with sleeping and eating. After having spent more time in their new families, the children seemed to become more relaxed and secure. Being more attached and feeling safe often permit children to show more of their emotionality toward their adoptive parents. The other two areas, activity and shyness, are more stable.

The adopted children's behaviour problems change during their stay in the adoptive family. After two years they show significantly more often physically challenging behaviour such as hitting, pushing, and throwing. At time of adoption the children were significantly less directly physically aggressive. Their challenging behaviour at that stage was more characterized by crying and being noisy and fussy. This kind of behaviour may be related to attention-seeking behaviour without annoying the parents too much. Being physically aggressive can be too challenging for the parents and can more easily provoke actions of rejection.

Children over 12 months at the time of adoption naturally score higher on communication and gross motor development. Children with a higher age at adoption also exhibited more challenging behaviour both physically and non-physically. These children were older at the time of measuring these variables, which logically explains the higher scores. It is more interesting to examine how gender and age at adoption influence the scores after two years. Age at adoption did not have a significant effect on communication at this stage. However, gender has an effect: girls score higher on communication which is in line with research on language development and gender [31]. Boys have higher scores on physically challenging behaviour. This has also been found in other studies [15, 32]. 

The paired sample *t*-test documented that the adopted children as a group had significantly increased their skills in communication and gross motor development. However, correlations between scores in both communication and gross motor skills after two years were not very high in contrast to temperamental characteristics and behaviour problems. The adopted children's communication and gross motor skills at age of adoption were weak predictors of later development in these areas. Since the children arrived at different age stages, their performance is strongly influenced by age at adoption. When controlling for age at adoption the correlation increased for both communication (.145 to  .368***) and gross motor development (.164 to  .251**) but not for temperamental characteristics and behaviour problems. These results indicate that age at adoption has an impact on the adopted children's general development. The children's temperamental traits and behaviour problems seem to be more stable during the first two years after adoption. 

Communicative competence is important for the development of internationally adopted children [33, 34]. At age of adoption communication in this study is measured by items covering areas such as participation in social play, responding to instructions, and pointing to an object. After two years two language expression and the correct use of “mine” and “yours” were also included. The adopted children had increased their communication competence from age at adoption to two years after adoption. However, two years on from adoption they still lag behind in communication compared to non-adopted children [35]. 

The regression analyses conducted at time of adoption and after two years using communication competence as the dependent variable showed that the independent variables explained more of the variance in the children's competence at time of adoption compared to two years later. The children arrived at different ages, so their performance at time of adoption obviously was influenced by this variable. Their gross motor development also had a significant impact on communication. After two years the variable age of adoption no longer had a significant impact on the children's communication. This is in line with conclusions from other studies showing that as adopted children grow older, their age at adoption explains less of the variation in their performance [16, 25]. It is interesting to see that the children's transition reaction has an impact on their communication after two years. Children with strong reactions had lower scores on the scale-measuring communication. Children that exhibit high frequency of physically challenging behaviour also had lower scores in communication. This behaviour may decrease when the children develop better language ability.

The hierarchical regressions conducted on different areas clearly documented that the adopted children's general development was hard to predict from their performance at time of adoption. Many of them have spent several months in institutions which make them vulnerable to delays in their physical and psychological development. At time of adoption their life circumstances change dramatically from one day to another. Being adopted involves meeting loving and caring parents who offer the child constant attention day and night. Many studies have documented dramatic changes in adopted children's development during the first months in their new families [6, 14, 33]. There can be considerable disparity among children at the time of adoption. However, most catch up with normal standards in weight, height, and motor development. 

Adopted children's communication and language development seem vulnerable to delays [16, 25, 36]. An institutional setting entails fewer opportunities for one-to-one contact with a stable caregiver and thus less chance of adequate language stimulation. Although most adopted children catch up in their language development at later age stages, around one third seems to acquire some language difficulties [14, 16, 37]. 

 The children's temperamental characteristics seem to be more stable and predictable. This is in line with outcomes from other studies [38, 39]. Children's temperamental dimensions have significant impact on their social interactions with parents and caregivers [40]. Furthermore, it is documented that these dimensions are included in attention disorders and development of behavior problems [41]. Adopted children with a difficult temperament in early childhood predict lower adjustment at age 7 in domains of social development, ego resilience, cognitive development, and behaviour problems [34]. Findings have indicated that individual temperament characteristics such as level of emotionality in toddler years have an impact on children's behaviour problems at later age stages [9]. High temperamental shyness and emotionality seem to be factors that to a certain extent predict internalizing behaviour problems [11, 42]. In our study, the adopted children's physically challenging behaviour had increased from age of adoption to two years later. This study will continue to follow up both temperamental characteristics and behaviour problems and their correlation with other significant factors at three to four years after adoption.

## 5. Conclusion

The results show that internationally adopted children are delayed in their general development when they first arrive in their adoptive families. Two years later the children have made significant progress in development. However, they still lag behind in communication and motor skills compared to non-adopted children. Temperamental characteristics seem quite stable from time of adoption until two years after adoption. Internationally adopted children exhibit low frequency of behaviour problems. However, the nature of the behaviour problems has changed during the two years. At time of adoption they show more non- physically challenging behaviour while two years later their physically challenging behaviour has increased.

## 6. Limitations

There are some limitations to the present study. The sample of the internationally adopted children was mostly collected from Eastern Norway, from a limited number of countries, and they had all been adopted before two years of age. These limitations make it difficult to generalize the results from the study to all internationally adopted children in Norway. Particularly important is the lack of older children in the sample. 

A further limitation is that all the information is based on interviews with parents (adoptive mothers). This may lessen the objectivity of the data, since the mothers are strongly involved with their children. However, here, it is important to mention that the data will be supplemented with more objective observations and interviews and with more external informants, such as kindergarten and school teachers, when the children become older. Furthermore, in this ongoing study the father will be the key informant three years after adoption. 

Since the present data form part of a larger longitudinal study following the target adopted children into early school years, there will be good future prospects of improving the limitations of the present study. 

Also, since the number of adopted children is quite small, the effect sizes in some of the analysis are rather small and may give somewhat weak basis for discovering differences within the sample. 

## Figures and Tables

**Table 1:** Gender, age of adoption, country of origin, and transition reactions witihin the sample (*N* = 112).

Gender	%	Age of adoption	%	Country of origin	%	Transition reactions at arrival	%
Girls	50.9	>12 months	58.9	China	30.4	No reactions	19.3
Boys	49.1	<12 months	41.1	South-Korea	19.6	Some reactions	62.4
				South-Africa	15.2	Great reactions	18.3
				Colombia	13.4		
				Ethiopia	10.7		
				Other countries	10.7		

**Table 2:** Differences in mean on general development (motor and communication), temperamental traits, (emotionality, activity, and shyness), and behavior problems (physically and non-physically challenging behavior) at time of adoption and at age two.

	Time of adoption (*N* = 102)			Age two (*N* = 101)			Cohen's *D* ^2^	*t*-value
	*M *	SD	*α* ^1^	*M*	SD	*α* ^1^
Motor and communication								
Ages and stages^3^								
Communication	29.22	16.60	.85	36.13	14.87	.86	.589	−3.385^∗∗∗ ^
Gross motor	36.67	19.16	.95	42.55	8.55	.56	.360	−3.163^∗∗∗^

Temperament								
EAS emotionality^4^	2.26	.73	.84	2.42	.70	.82	.310	−2.834^∗∗∗^
EAS activity level	3.85	.66	.76	3.76	.71	.84	−.193	1.147
EAS shyness	2.33	.70	.76	2.36	.61	.73	.061	−.396

Behavior problems^5^								
Physical challenging behavior	2.03	1.06	.54	2.23	.99	.81	.267	−1.983^∗^
Non-physically challenging behavior	3.08	1.26	.76	2.61	.84	.84	.528	4.109^∗∗∗^

Note. ^1^Reliability coefficient (Cronbach's alpha), ^2^effect size (Cohen's *D*), ^3^high score is positive, ^4^high score indicate higher frequencies, and ^5^high score indicate higher frequencies.

^∗∗∗^ *P* < .001, ^∗∗^ *P* < .01, ^∗^ *P* < .05.

**Table 3:** Correlation and partial correlation between scores at time of adoption and at age two.

	Correlation *r *	Partial correlation^1^ *r*_ab.c_
Communication	.145	.368^∗∗∗^
Gross motor	.164	.251^∗∗^
Physically chal. behavior	.471^∗∗∗^	.501^∗∗∗^
Non-physically chal. B.	.432^∗∗∗^	.496^∗∗∗^
Emotionality	.688^∗∗∗^	.691^∗∗∗^
Activity	.430^∗∗^	.462^∗∗∗^
Shyness	.517^∗∗∗^	.507^∗∗∗^

Note. ^1^Controlling for age at adoption.

^∗∗∗^ *P* < .001, ^∗∗^ *P* < .01, ^∗^ *P* < .05.

**Table 4:** Linear regression analysis at time of adoption and at age two: communication as dependent variable.

	At time of adoption	At age two
	*β*	*β*
Age at adoption	.270^∗∗^	−.152
Gender	−.191^∗∗^	−.089^∗^
Transition reactions	−.093	−.144^∗^
Kina/Korea	.021	−.161
Colombia/others	.070	−.207
Gross motor development	.457^∗∗∗^	.357^∗∗∗^
Emotionality	.015	.001
Activity	−.011	−.089
Shyness	.099	.138
Physically challenging behavior	−.072	−.316^∗∗^
Non-physical chal. behavior	.172	.094

	*R* ^2^ = .650 *F*(11,91) = 15.375***	*R* ^2^ = .381 *F*(11,94) = 5.262***

Note. Dependent variable: communication at age two.

^∗∗∗^ *P* < .001, ^∗∗^ *P* < .01, ^∗^ *P* < .05.

**Table 5:** Hierarchical multiple regression analysis predicting communication, temperamental characteristics, and behavior problems at age two (*N* = 100).

	Δ*R* ^2^	**β**	*R* ^2^
*Communication at age two*			
Step 1	.02		
Communication (at adoption)		.15	
Step 2^1^	.25^∗∗^		.27^∗∗∗^
Gender		−.22^∗^	
Transition reactions		−.18^∗^	
Age at adoption		−.45^∗∗^	

*Gross motor development at age two*			
Step 1	.02		.10^∗∗∗^
Gross motor development (at adoption)		.15	
Step 2^1^	.08		

*Emotionality at age two*			
Step 1	.47^∗∗∗^		.51^∗∗∗^
Emotionality (at adoption)		.69^∗∗∗^	
Step 2^1^	.04		

*Activity at age two*			
Step 1	.19^∗∗∗^		.50^∗∗∗^
Activity (at adoption)		.43^∗∗∗^	
Step 2^1^	.07		

*Shyness at age two*			
Step 1	.27^∗∗∗^		.32
Shyness (at adoption)		.52^∗∗∗^	
Step 2^1^	.06		

*Physically challenging behavior at age two*			
Step 1	.22^∗∗∗^		
Physically chal. Behave (at adoption)		.47^∗∗∗^	.35^∗∗∗^
Step 2^1^	.13		
Gender		.18^∗^	

*Non-physically challenging behavior at age two*			
Step 1	.19^∗∗∗^		
Non-physical Chal. behavior		.43^∗∗∗^	.26
Step 2^1^	.08		
Age at adoption		−.25^∗^	

Note. ^1^Independend variables: gender, transition reactions, and age of adoption. Only significant values are presented. ^∗∗∗^ *P* < .001, ^∗∗^ *P* < .01, and ^∗^ *P* < .05.
